# Potential antiviral effects of some native Iranian medicinal plants extracts and fractions against influenza A virus

**DOI:** 10.1186/s12906-021-03423-x

**Published:** 2021-10-01

**Authors:** Parvaneh Mehrbod, Hanieh Safari, Zeinab Mollai, Fatemeh Fotouhi, Yasaman Mirfakhraei, Hanieh Entezari, Saied Goodarzi, Zahra Tofighi

**Affiliations:** 1 Influenza and Respiratory Viruses Department, Pasteur Institute of IRAN, Tehran, Iran; 2 Department of Pharmacognosy, Faculty of Pharmacy, Tehran University of Medical Sciences, Tehran, Iran; 3 Medicinal Plants Research Center, Faculty of Pharmacy, Tehran University of Medical Sciences, Tehran, Iran

**Keywords:** Licorice, Lemon balm, White willow, Myrtle, Black tea, Anti-influenza A viral activity

## Abstract

**Background:**

Influenza A virus (IAV) infection is a continual threat to the health of animals and humans globally. Consumption of the conventional drugs has shown several side effects and drug resistance. This study was aimed to screen some Iranian medicinal plants extracts and their fractions against influenza A virus.

**Methods:**

*Glycyrrhiza glabra* (rhizome), *Myrtus  commonis* (leaves), *Melissa officinalis* (leaves), *Hypericum perforatum* (aerial parts), *Tilia platyphyllos* (flower), *Salix alba* (bark), and *Camellia sinensis* (green and fermented leaves) were extracted with 80% methanol and fractionated with chloroform and methanol, respectively. The cytotoxicity of the compounds were determined by MTT colorimetric assay on MDCK cells. The effective concentrations (EC_50_) of the compounds were calculated from the MTT results compared to the negative control with no significant effects on cell viability. The effects of EC_50_ of the compounds on viral surface glycoproteins and viral titer were tested by HI and HA virological assays, respectively and compared with oseltamivir and amantadine. Preliminary phytochemical analysis were done for promising anti-IAV extracts and fractions.

**Results:**

The most effective samples against IAV titer (*P* ≤ 0.05) were crude extracts of *G. glabra*, *M. officinalis* and *S. alba*; methanol fractions of *M. communis* and *M. officinalis*; and chloroform fractions of *M. communis* and *C. sinensis* (fermented) mostly in co- and pre-penetration combined treatments. The potential extracts and fractions were rich in flavonoids, tannins, steroids and triterpenoids.

**Conclusion:**

The outcomes confirmed a scientific basis for anti-influenza A virus capacity of the extracts and fractions from the selected plants for the first time, and correlated their effects with their phytochemical constituents. It is worth focusing on elucidating pure compounds and identifying their mechanism(s) of action.

**Supplementary Information:**

The online version contains supplementary material available at 10.1186/s12906-021-03423-x.

## Background

Influenza A virus (IAV) is one of the most severe respiratory diseases which leads to the high rates of morbidity and mortality [1, 2]. Vaccination, as a peventive method, can not provide sufficient control against the spread of this infection because of continuous antigenic drifts [3]. The adamantane derivatives including amantadine and rimantadine were used for the treatment and prophylaxis of influenza disease for several years [4]. Oseltamivir (Tamiflu®), as FDA-approved drug, which is a neuraminidase inhibitor molecule with the basic structure of shikimic acid was isolated and purified from the fruits of *Illicium verum* Hook. f. (Illiciaceae) [5]. Treatment with conventional drugs including amantadine and oseltamivir has shown side effects on the central nervous system and the gastrointestinal tract. Also, because of the genetic instability and reassortments of the virus from one side and drug resistance from the other side, the prescription of these drugs has been inefficient in some cases [6–8]. Due to the need for having other inhibitors of IAV, the scientists have focused on the screening medicinal plants and natural products and to find and introduce a lead compound or structure for the further preclinical trials [9].

Medicinal plants play vital roles in prevention and treatment of several diseases. The natural products and medicinal plants are popular in different parts of the world from developing to developed countries as complementary to common medicines [10, 11]. Medicinal plants are cheaper, more available and culturally acceptable especially in Iran with a strong and long history of traditional medicine in comparison with some chemical drugs with undesirable adverse effects [12].

There are several plants used from ancient times till now for the treatment of common cold, flu syndrom and infections in throat and upper respiratory systems. Although there were many ethnopharmacology articles about them, a few reports highlighted their anti-influenza virus effects [13, 14].

To the best of our knowledge, there are no reports about antiviral effects of *G. glabra*, *M. commonis*, *S. alba*, *T. platyphyllos* and *C. sinensis* (fermented) extracts against influenza A virus. Also, there are no reports about anti-influenza effects of fractions of other mentioned plants. The objective of this study was to investigate the potential effects of crude extracts and different fractions of some Iranian medicinal plants against influenza replication, and compare their activities with oseltamivir and amantadine as control drugs. For this purpose, the propagation and cytopathic effects of IAV in the presence of different extracts and fractions were determined using the hemagglutination (HA), hemagglutination inhibition (HI), and MTT cytotoxicity assays. The potent extract(s) and fraction(s) can be introduced as sources of phytochemicals with antiviral effects against influenza A virus.

## Methods

### Preparation of plant extracts and fractions

Rhizomes of *Glycyrrhiza glabra* (Leguminosae, PMP-246), leaves of *Myrtus commonis* (Myrtaceae, PMP-421) and *Melissa officinalis* (Labiatae, PMP-358), aerial parts of *Hypericum perforatum* (Hypericaceae, PMP-526), and bark of *Salix alba* (Salicaceae, PMP-924) were purchased in July 2017 from market in Tehran, Iran. Dried and fermented leaves of *Camellia sinensis* (Theaceae, PMP-415 and PMP-416, respectively) were obtained in November 2017 from market of Lahijan, Iran. Flowers of *Tilia platyphyllos* (Malvaceae, 7057-TEH) were collected from Ramsar, Mazandaran, Iran. The voucher speciments were identified by Dr. Zahra Tofighi and Dr. Yousef Ajani, and deposited in herbarium of Faculty of Pharmacy, Tehran University of Medical Sciences, Tehran, Iran. All dried plants were extracted with 80% methanol and fractionated with chloroform to give chloroform fractions and the residues were named methanol fractions.

### Cell culture and influenza virus propagation

Madin Darby Canine Kidney (MDCK) cells were maintained in Dulbecco’s Modified Eagle’s Medium (DMEM) (Mediatech Cellgro, USA) containing 10% Fetal Bovine Serum (FBS) (PAA, Austria) and 1% Penicillin and streptomycin (Mediatech Cellgro, USA) in 5% CO_2_ incubator at 37 °C. Influenza virus strain [A/Puerto Rico/8/1934 (H1N1) (ATCC VR-1469™)] was prepared from Influenza Department, Pasteur Institute of Iran, and propagated in MDCK cells. DMEM supplemented with 1 μg/ml of Trypsin-TPCK (Tosylamide Phenylethyl Chloromethyl Keton-treated Trypsin) (Sigma, USA) was used as maintenance medium during antiviral experiments. For measuring the virus infectivity dose, the 50% cell culture infectious dose (CCID_50_) was obtained by the hemagglutination assay using Karber method [15, 16].

### Cytotoxicity assay

Two-fold serial dilutions of the extracts and fractions were exposed to the sub-cultured MDCK cells in 96-well plates (3 × 10^4^ cell/well), for 48 h at 37 °C in duplicates. The test was repeated two times. The colorimetric MTT assay was performed as previously described by Mehrbod et al. [16]. Briefly, the culture medium was replaced by MTT 1X [3-(4,5-dimethyl-2-thiazolyl)-2,5-diphenyl-2H-tetrazolium bromide; Sigma, USA]. Following 3–4 h incubation at 37 °C in dark, DMSO (100 μl) was added to each well to release the purple color of formazan. The absorbance at 570 nm was measured with a microplate reader (BioTek EL 800, US). The cell viability was calculated based on the following formula: (mean Optical Density (OD) of treated cells/mean OD of control cells) × 100. The 50% cytotoxic concentration (CC_50_), the concentration that reduced the cell viability by 50% respect to the control cells; and the 50% effective concentration (EC_50_), the concentration required to achieve 50% protection against virus induced cytopathic effect, were also calculated by analyzing MTT data using SPSS software. The cells without any exposure which were considered as negative controls and DMSO as a vehicle control with maximum 0.5% concentration were tested in parallel. Amantadine hydrochloride and oseltamivir carboxylate, the conventional antiviral compounds, were also tested as positive control drugs.

### Selectivity index

The selectivity index (SI) calculated by dividing CC_50_ to EC_50_, represents the relative safety of the extracts or frections. It has been demonstrated that compounds with selectivity indices higher than 3 are potentially safe antiviral reagents [17].

### Antiviral assay

In co-penetration procedure of antiviral evaluations, influenza virus (100 TCID_50_/0.1 ml) was mixed with the EC_50_ of the extracts and fractions for 30 min, and then incubated with the cells at 37 °C. In pre-penetration and post-penetration procedures, the virus was added to the cells after and before the extracts and fractions. Following 1 h incubation, the unabsorbed viruses were replaced by TPCK-containing medium (1 μg/ml). Following 48 h incubation at 37 °C, viabilities of the cells were measured by MTT assay as described earlier. Concurrently, the virus titer was determined by testing the cell supernatants using the HA assay [16]. Amantadine hydrochloride (98.5 μg/ml) and oseltamivir carboxylate (394.25 μg/ml) (Sigma, Saint Louis, Missouri, USA) were considered as positive control drugs and the cells without any exposure were tested as negative controls. DMSO was tested as a vehicle control with maximum 0.5% concentration. The test was repeated two times in duplicates.

### Cellular percentage of protection

The protection percentages of the samples were calculated by SPSS from the MTT data results of mock-infected and infected cells after 48 h exposure, using the following formula: Percentage of protection = [(ODT) V − (ODC) V] / [(ODC)M − (ODC) V] × 100 where (ODT)V, (ODC)V and (ODC)M represent the absorbance of the treated sample, the virus-infected control (no compound) and the negative control (mock), respectively [18].

### Hemagglutination assay (HA)

For quantification of the virus titer from the cell supernatants, the HA assay was conducted as previously described [19]. Briefly, serial dilutions of the culture media were added to 96-well U-shaped microplates in duplicates. The test was repeated two times. The HA units were measured as the reciprocal of the highest dilution giving complete agglutination with chicken red blood cells (0.5%). The precipitation and diffuse lattice formation of the RBCs demonstrate the absence and the presence of the virus, respectively.

### Hemagglutination inhibition assay (HI)

For investigation of the inhibitory effect of the samples on the hemagglutinin activity, the concentration of 4 HA unit of virus particles was used. Briefly, the extracts and fractions were diluted 2-fold serially from CC_50_ concentration. Then, 4 HA unit of the virus was added to each well. After pre-incubation for 45 min at room temperature, chicken red blood cells (0.5%) were added to the solution. The physical interaction between extracts/fractions and virus surface HA glycoprotein was calculated after 1 h by the agglutination inhibition pattern.

### Preliminary phytochemical analysis

Crude extracts, chloroform and methanol fractions of the effective anti-influenza plants were tested for identifying the class of active metabolites such as alkaloids, cardiac glycosides, tannins, flavonoids, steroids, triterpenoids and saponins by following the standard procedures [20, 21].

#### Tests for alkaloids

A) Dragendorff’s test: The apperance of reddish brown turbidity after addition of Dragendorff reagent was indicator for the presence of alkaloids.

B) Hager’s test*:* By addition of Hager reagent to the samples, yellow turbidity was seen in the presence of alkaloids.

#### Tests for cardiac glycosides

Keller-killiani test: The mixture of ferric chloride and glacial acetic acid were added to the sample solutions. Apperance of bluish green in the upper layer and reddish color in the lower layer of the solutions, after addition of concentrated sulphuric acid confirmed the existence of cardiac glycosides in the samples.

#### Test for tannins

FeCl_3_ test: After adding a few drops of 5% w/v solution of ferric chloride III in 90% alcohol to the sample solutions, the appearance of deep blue or dark green color indicated the tannin existence in the samples.

#### Test for flavonoids (Shinoda test)

After adding few drops of concentrated HCl and magnesium metal to the samples, the appearance of pink, red, crimson or magenta color was the sign of flavonoids presentation.

#### Tests for steroids and triterpenoids

Libermann-Buchard test: The appearance of brown ring in the middle of green upper layer and deep red lower layer after addition of sulfuric acid containing few drops of acetic anhydride were indicator for the presence of steroids and triterpenoids.

Salkowski test: By addition of chloroform and concentrated H_2_SO_4_ to the extract and shaking well, the color of chloroform layer changed to red, and acid layer showed greenish yellow fluorescent if sterols and/or triterpenes existed.

#### Test for saponins (foam test)

The volume of 50 mg of samples in water was shaken vigorously in a test tube. If froth characteristic was obtained, the presence of saponins was confirmed.

### Statistical analysis

The data were expressed as mean ± SD, and analyzed by one-way analysis of variance (ANOVA) and General Linear Model (GLM) (SPSS 18.0), Tukey and Duncan post-hoc tests. Sample values with *P* ≤ 0.05 and *P* ≤ 0.01 were considered statistically significant and highly significant, respectively.

## Results

In this study, the efficacy of the crude extracts, chloroform and methanol fractions of 8 selected plants with history of usages in traditinal and folklore Iranian medicine were tested against IAV. The cytotoxicity of the extracts were evaluated, and non-toxic concentrations were defined prior to antiviral assay. The ability of the samples against viral titer and viral cytopathic effects varied with different extracts and fractions of the same plant as determined by the HA and MTT assays. The profile of the extracts and fractions of selected medicinal plants used in this study is listed in Table 1.

**Table 1:** Profile of the medicinal plants extraction

Botanical Name	Extract yeilds (%)	Chl F. Yeild (%)	Met F. Yeild (%)
*Glycyrrhiza glabra*	27.2	16.4	75.9
*Myrtus communis*	39.4	19.8	72.9
*Melissa officinalis*	42.2	16.5	79.8
*Hypericum perforatum*	39.1	28.0	66.2
*Tilia platyphyllos*	11.9	41.5	55.3
*Salix alba*	15.5	6.6	57.1
*Camellia sinensis*	36.8	55.4	41.4
*Camellia sinensis* (fermented)	25.0	40.0	60.0

*Chl F*. Chloroform Fraction, *Met F*. Methanol Fraction

### Cytotoxicity and selectivity indices of the compounds

The results for CC_50_ were largely different for various samples (Table 2). Among the crude extracts, the highest CC_50_ value belonged to *S. alba* (3647.45 ± 52.52 μg/ml) and the lowest CC_50_ value (10.41 ± 0.00 μg/ml) was obtained for *M. communis* and *H. perforatum* equally. Among the chloroform fractions, *S. alba* and *M. communis* showed the highest (1755.28 ± 0.89 μg/ml) and the lowest (2.60 ± 0.00 μg/ml) CC_50_ values, respectively. Amongst methanol fractions the highest and the lowest CC_50_ values were obtained for *M. officinalis* (4413.80 ± 3.69 μg/ml) and *T. platyphyllos* (277.56 ± 3.19 μg/ml), respectively. The EC_50_ values which were the same as non-cytotoxic concentrations (NCTC) with no significant effects on the cell viability were calculated using data obtained from MTT and one-way ANOVA analysis as compared to the negative control (Table 2). The selectivity index values were obtained with the highest SI value of 32 with *M. officinalis* (chloroform fraction), *H. perforatum* (methanol fraction), *S. alba* (crude extract and methanol fraction) and *C. sinensis* unfermented (methanol fraction) and the lowest value of 2 with *G. glabra* (chloroform fraction).

**Table 2:** Anti-influenza A virus effects of some medicinal plants extracts and fractions

Plant	Extracts or Fractions	Concentration in DMSO	CC_**50**_ (μg/ml)	NCTC (μg/ml) & EC_**50**_ (μg/ml)	Selectivity index (SI = CC_**50**_/EC_**50**_)
***Glycyrrhiza glabra***	Crude E.	100 mg/ml	881.87 ± 0.03	55.12	16
***Glycyrrhiza glabra***	Chloroform F.	100 mg/ml	5.19 ± 0.00	2.57	2
***Glycyrrhiza glabra***	Methanol F.	100 mg/ml	3529.48 ± 0.76	441.12	8
***Myrtus communis***	Crude E.	100 mg/ml	10.40 ± 0.00	2.60	4
***Myrtus communis***	Chloroform F.	100 mg/ml	2.60 ± 0.00	0.65	4
***Myrtus communis***	Methanol F.	100 mg/ml	603.67 ± 0.60	37.73	16
***Melissa officinalis***	Crude E.	100 mg/ml	1813.99 ± 0.23	113.37	16
***Melissa officinalis***	Chloroform F.	100 mg/ml	1427.10 ± 0.45	44.62	32
***Melissa officinalis***	Methanol F.	100 mg/ml	4413.80 ± 3.69	551.72	8
***Hypericum perforatum***	Crude E.	100 mg/ml	10.41 ± 0.00	1.30	8
***Hypericum perforatum***	Chloroform F.	100 mg/ml	10.99 ± 0.00	2.75	4
***Hypericum perforatum***	Methanol F.	100 mg/ml	3929.08 ± 2.47	122.7	32
***Tilia platyphyllos***	Crude E.	100 mg/ml	152.26 ± 0.50	9.56	15.92
***Tilia platyphyllos***	Chloroform F.	100 mg/ml	267.45 ± 8.8	16.69	16.03
***Tilia platyphyllos***	Methanol F.	100 mg/ml	277.56 ± 3.19	34.62	8.02
***Salix alba***	Crude E.	100 mg/ml	3647.45 ± 52.52	113.70	32
***Salix alba***	Chloroform F.	100 mg/ml	1755.28 ± 0.89	219.37	8
***Salix alba***	Methanol F.	100 mg/ml	2816.86 ± 77.80	88.03	32
***Camellia sinensis***	Crude E.	100 mg/ml	161.89 ± 2.30	20.25	8
***Camellia sinensis***	Chloroform F.	100 mg/ml	13.28 ± 0.00	1.62	8.17
***Camellia sinensis***	Methanol F.	100 mg/ml	877.28 ± 3.80	27.41	32
***Camellia sinensis*)fermented(**	Crude E.	100 mg/ml	1038.192 ± 88.73	129.87	8
***Camellia sinensis*)fermented(**	Chloroform F.	100 mg/ml	875.61 ± 7.55	109.50	8
***Camellia sinensis*)fermented(**	Methanol F.	100 mg/ml	405.13 ± 3.71	50.62	8
**Amantadine hydrochloride**	**–**	2000 μg/ml H_2_O	197.001 ± 1.533	98.500	2
**Oseltamivir carboxylate**	**–**	4000 μg/ml H_2_O	788.505 ± 6.006	394.250	2

*CC*_*50*_ 50% cytotoxic concentration, *EC*_*50*_ 50% effective concentration, *NCTC* non-cytotoxic concentrations (EC_50_ and NCTC values were equal), *SI* Selectivity Index

### Inhibitory effect of extracts and fractions on influenza a virus

The samples were tested in an in vitro screening assay to define the antiviral activity against IAV. The antiviral activity of the plants extracts/fractions was analyzed based on the Log_10_ HA titer (Table 3) and Log HA decrement (Table 4). Among them, in co-penetration treatments, *G. glabra* (crude extract; 5.00 ± 4.24), *G. glabra* (methanol fraction; 5.50 ± 3.54), *M. communis* (chloroform fraction; 5.50 ± 3.54) and *M. officinalis* (methanol fraction;6.00 ± 2.83); in pre-penetration treatment, *M. communis* (methanol fraction; 5.50 ± 0.71); and in post-penetration treatment: *M. communis* (chloroform fraction; 5.50 ± 3.54) showed highly significant (*P* ≤ 0.01) decrease in HA titer compared to the virus inoculation. These results were in accordance with the Log HA decrement (Table 5) which showed 5–6 logs decrement in Log HA titer for all samples.

**Table 3:** Log_10_ HA titer from HA assay in combined treatments with virus as compared to virus control group

Plant	Extracts or Fractions	Log HA (mean ± SD)		
		Co-pen	Pre-pen	Post-pen
***Glycyrrhiza glabra***	Crude E.	0.90 ± 1.28^**^	1.20 ± 0.43^*^	1.50 ± 0.00
***Glycyrrhiza glabra***	Chloroform F.	2.11 ± 0.00	2.11 ± 0.00	1.81 ± 0.43
***Glycyrrhiza glabra***	Methanol F.	0.75 ± 1.06^**^	1.96 ± 0.21	2.11 ± 0.00
***Myrtus communis***	Crude E.	1.66 ± 0.21	1.66 ± 0.21	1.66 ± 0.21
***Myrtus communis***	Chloroform F.	0.75 ± 1.06^**^	1.20 ± 0.43^*^	0.75 ± 1.06^**^
***Myrtus communis***	Methanol F.	1.50 ± 0.43	0.75 ± 0.21^**^	1.20 ± 0.00^*^
***Melissa officinalis***	Crude E.	1.35 ± 0.21^*^	1.96 ± 0.21	2.11 ± 0.00
***Melissa officinalis***	Chloroform F.	1.66 ± 0.21	1.66 ± 0.21	1.66 ± 0.21
***Melissa officinalis***	Methanol F.	0.60 ± 0.85^**^	2.11 ± 0.00	1.96 ± 0.21
***Hypericum perforatum***	Crude E.	1.50 ± 0.43	1.81 ± 0.00	2.11 ± 0.00
***Hypericum perforatum***	Chloroform F.	1.66 ± 0.21	1.81 ± 0.01	1.66 ± 0.21
***Hypericum perforatum***	Methanol F.	1.50 ± 0.43	1.66 ± 0.21	1.50 ± 0.43
***Tilia platyphyllos***	Crude E.	1.81 ± 0.00	1.65 ± 0.21	1.50 ± 0.00
***Tilia platyphyllos***	Chloroform F.	1.50 ± 0.43	1.66 ± 0.21	1.50 ± 0.00
***Tilia platyphyllos***	Methanol F.	1.20 ± 0.00^*^	1.96 ± 0.21	1.81 ± 0.00
***Salix alba***	Crude E.	1.20 ± 0.43^*^	1.35 ± 0.21^*^	1.20 ± 0.43^*^
***Salix alba***	Chloroform F.	1.35 ± 0.21^*^	1.81 ± 0.43	1.35 ± 0.21^*^
***Salix alba***	Methanol F.	1.50 ± 0.43	1.65 ± 0.64	1.65 ± 0.64
***Camellia sinensis***	Crude E.	2.26 ± 0.2	1.96 ± 0.21	1.96 ± 0.21
***Camellia sinensis***	Chloroform F.	1.81 ± 0.000	1.96 ± 0.21	2.11 ± 0.43
***Camellia sinensis***	Methanol F.	2.26 ± 0.21	2.26 ± 0.21	1.50 ± 0.00
***Camellia sinensis*(fermented)**	Crude E.	1.65 ± 0.21	1.81 ± 0.00	1.66 ± 0.21
***Camellia sinensis*(fermented)**	Chloroform F.	1.20 ± 0.43^*^	1.35 ± 0.64^*^	1.35 ± 0.64^*^
***Camellia sinensis*(fermented)**	Methanol F.	1.50 ± 0.00	1.81 ± 0.00	1.81 ± 0.00
**Amantadine hydrochloride**	**–**	0.30 ± 0.00^**^	2.11 ± 0.00	2.11 ± 0.00
**Oseltamivir carboxylate**	**–**	0.00 ± 0.00^**^	0.00 ± 0.00^**^	0.00 ± 0.00^**^
**Influenza virus**	**–**	2.41 ± 0.00	2.41 ± 0.00	2.41 ± 0.00

Data presented as mean ± SD are averages of two independent HA titration which were tested in duplicates. *, **: Significantly and highly significantly different from values obtained for compound-treated samples compared to untreated sample (*P* ≤ 0.05 & *P* ≤ 0.01) analyzed by SPSS, Tukey post-hoc test

**Table 4:** Log HA decrement obtained from HA assay

Plant	Extracts or Fractions	Log HA decrement (mean ± SD)		
		Co-pen	Pre-pen	Post-pen
***Glycyrrhiza glabra***	Crude E.	5.00 ± 4.24^abc^	4.00 ± 1.41^bc^	3.00 ± 0.00^abc^
***Glycyrrhiza glabra***	Chloroform F.	1.00 ± 0.00^a^	1.00 ± 0.00^a^	2.00 ± 1.41^ab^
***Glycyrrhiza glabra***	Methanol F.	5.50 ± 3.54^abc^	1.50 ± 0.71^a^	1.00 ± 0.00^a^
***Myrtus communis***	Crude E.	2.50 ± 0.71^ab^	2.50 ± 0.71^ab^	2.50 ± 0.71^ab^
***Myrtus communis***	Chloroform F.	5.50 ± 3.54^abc^	4.00 ± 1.41^bc^	5.50 ± 3.54^c^
***Myrtus communis***	Methanol F.	3.00 ± 1.41^abc^	5.50 ± 0.71^c^	4.00 ± 0.00^bc^
***Melissa officinalis***	Crude E.	3.50 ± 0.71^abc^	1.50 ± 0.71^a^	1.00 ± 0.00^a^
***Melissa officinalis***	Chloroform F.	2.50 ± 0.71^ab^	2.50 ± 0.71^ab^	2.50 ± 0.71^ab^
***Melissa officinalis***	Methanol F.	6.00 ± 2.83^abc^	1.00 ± 0.00^a^	1.50 ± 0.71^ab^
***Hypericum perforatum***	Crude E.	3.00 ± 1.41^abc^	2.00 ± 0.00^a^	1.00 ± 0.00^a^
***Hypericum perforatum***	Chloroform F.	2.50 ± 0.71^ab^	2.00 ± 0.00^a^	2.50 ± 0.71^ab^
***Hypericum perforatum***	Methanol F.	3.00 ± 1.41^abc^	2.50 ± 0.71^ab^	3.00 ± 1.41^abc^
***Tilia platyphyllos***	Crude E.	2.00 ± 0.00^ab^	2.00 ± 0.00^abc^	3.00 ± 0.00^abc^
***Tilia platyphyllos***	Chloroform F.	3.00 ± 1.41^b^	2.50 ± 0.71^abc^	3.00 ± 0.00^abc^
***Tilia platyphyllos***	Methanol F.	4.00 ± 0.00^b^	4.00 ± 0.00^c^	2.00 ± 0.00^abc^
***Salix alba***	Crude E.	4.00 ± 1.41^b^	3.50 ± 0.71^bc^	4.00 ± 1.41^c^
***Salix alba***	Chloroform F.	3.50 ± 0.71^b^	2.00 ± 1.41^abc^	3.50 ± 0.71^bc^
***Salix alba***	Methanol F.	3.00 ± 1.41^b^	2.50 ± 2.12^abc^	2.50 ± 2.12^abc^
***Camellia sinensis***	Crude E.	0.50 ± 0.71^a^	1.50 ± 0.71^ab^	1.50 ± 0.71^abc^
***Camellia sinensis***	Chloroform F.	2.00 ± 0.00^ab^	2.50 ± 0.71^ab^	1.00 ± 1.41^ab^
***Camellia sinensis***	Methanol F.	0.50 ± 0.71^a^	0.50 ± 0.71^a^	3.00 ± 0.00^abc^
***Camellia sinensis*(fermented)**	Crude E.	2.50 ± 0.71^ab^	2.00 ± 0.00^abc^	2.50 ± 0.71^abc^
***Camellia sinensis*(fermented)**	Chloroform F.	4.00 ± 1.41^b^	3.50 ± 2.121^bc^	3.50 ± 2.12^bc^
***Camellia sinensis*(fermented)**	Methanol F.	3.00 ± 0.00^b^	2.00 ± 0.00^abc^	2.00 ± 0.00^abc^
**Amantadine hydrochloride**	**–**	7.00 ± 0.00^bc^	1.00 ± 0.00^a^	1.00 ± 0.00^a^
**Oseltamivir carboxylate**	**–**	8.00 ± 0.00^c^	8.00 ± 0.00^d^	8.00 ± 0.00^d^

Data presented as mean ± SD are averages of two independent HA titration which were tested in duplicates. Different letters show significant differences in each column (Duncan Grouping)

**Table 5:** Cell viabilities from MTT assay in combined treatments with virus as compared to virus control group

Plant	Extracts or Fractions	Cell viability (mean ± SD)		
		Co-pen	Pre-pen	Post-pen
***Glycyrrhiza glabra***	Crude E.	0.42 ± 0.04^*^	1.05 ± 0.10^**^	0.41 ± 0.11^**^
***Glycyrrhiza glabra***	Chloroform F.	0.21 ± 0.03	0.21 ± 0.03	0.43 ± 0.04^**^
***Glycyrrhiza glabra***	Methanol F.	0.86 ± 0.29^**^	0.71 ± 0.10^**^	0.36 ± 0.11^**^
***Myrtus communis***	Crude E.	0.49 ± 0.01^**^	0.54 ± 0.05^**^	0.8 ± 0.05^**^
***Myrtus communis***	Chloroform F.	0.77 ± 0.11^**^	0.49 ± 0.04^**^	0.42 ± 0.11^**^
***Myrtus communis***	Methanol F.	0.20 ± 0.01	0.56 ± 0.10^**^	0.37 ± 0.01^**^
***Melissa officinalis***	Crude E.	0.50 ± 0.04^**^	0.212 ± 0.02	0.29 ± 0.10
***Melissa officinalis***	Chloroform F.	0.21 ± 0.03	1.01 ± 0.31^**^	0.32 ± 0.04
***Melissa officinalis***	Methanol F.	0.81 ± 0.05^**^	0.45 ± 0.06^*^	0.84 ± 0.09^**^
***Hypericum perforatum***	Crude E.	0.51 ± 0.03^**^	0.35 ± 0.01	0.40 ± 0.04^**^
***Hypericum perforatum***	Chloroform F.	0.62 ± 0.12^**^	0.62 ± 0.11^**^	0.91 ± 0.04^**^
***Hypericum perforatum***	Methanol F.	0.21 ± 0.03	0.66 ± 0.08^**^	0.79 ± 0.06^**^
***Tilia platyphyllos***	Crude E.	0.25 ± 0.02	0.21 ± 0.01	0.52 ± 0.16^**^
***Tilia platyphyllos***	Chloroform F.	0.72 ± 0.04^**^	0.34 ± 0.07	0.68 ± 0.07^**^
***Tilia platyphyllos***	Methanol F.	0.81 ± 0.05^**^	0.50 ± 0.02^**^	0.47 ± 0.12^**^
***Salix alba***	Crude E.	0.80 ± 0.07^**^	0.73 ± 0.03^**^	0.533 ± 0.08^**^
***Salix alba***	Chloroform F.	0.87 ± 0.11^**^	0.63 ± 0.05^**^	0.546 ± 0.01^**^
***Salix alba***	Methanol F.	0.66 ± 0.07^**^	0.50 ± 0.03	0.420 ± 0.07
***Camellia sinensis***	Crude E.	0.29 ± 0.01	0.39 ± 0.09	0.582 ± 0.075^**^
***Camellia sinensis***	Chloroform F.	0.74 ± 0.13^**^	0.81 ± 0.16^**^	0.385 ± 0.076
***Camellia sinensis***	Methanol F.	0.29 ± 0.03	0.26 ± 0.01	0.698 ± 0.11^**^
***Camellia sinensis*(fermented)**	Crude E.	0.87 ± 0.11^**^	0.74 ± 0.13^**^	0.581 ± 0.03^**^
***Camellia sinensis*(fermented)**	Chloroform F.	0.81 ± 0.12^**^	0.79 ± 0.26^**^	0.468 ± 0.08^**^
***Camellia sinensis*(fermented)**	Methanol F.	0.80 ± 0.05^**^	0.59 ± 0.02^**^	0.555 ± 0.04^**^
**Amantadine hydrochloride**	**–**	1.01 ± 0.03^**^	0.59 ± 0.05^**^	0.61 ± 0.06^**^
**Oseltamivir carboxylate**	**–**	1.11 ± 0.01^**^	1.06 ± 0.01^**^	1.04 ± 0.03^**^
**Influenza virus**		0.15 ± 0.05	0.15 ± 0.05	0.15 ± 0.02

Data presented as mean ± SD are averages of two independent MTT assays which were tested in duplicates. **: highly significantly different from values obtained for drugs-treated samples compared to untreated sample (*P* ≤ 0.01) analyzed by SPSS, Tukey post-hoc test

### Cell viability and cellular percentage of protection

The optical densities (ODs) in MTT assay for the antiviral activity against IAV revealed highly significant increments in the majority of the combined treatments compared to the virus-inoculated cells (*P* ≤ 0.01). Amantadine and oseltamivir combined exposures also resulted in high cell viability (*P* ≤ 0.01) (Table 5). The ODs in combined treatments were analyzed to obtain the percentage of compounds protection on the cell viability against the virus infectivity. Data are presented in Table 6.

**Table 6:** Cellular percentage of protection in combined treatments with virus as compared to control groups

Plant	Extracts or Fractions	Percentage of protection (mean ± SD)		
		Co-pen	Pre-pen	Post-pen
***Glycyrrhiza glabra***	Crude E.	24.75 ± 3.88	88.99 ± 10.53	23.02 ± 10.91
***Glycyrrhiza glabra***	Chloroform F.	3.24 ± 3.20	3.33 ± 3.38	25.78 ± 4.40
***Glycyrrhiza glabra***	Methanol F.	69.50 ± 29.42	53.93 ± 10.02	18.77 ± 10.75
***Myrtus communis***	Crude E.	31.52 ± 0.34	36.67 ± 4.95	71.06 ± 4.94
***Myrtus communis***	Chloroform F.	60.04 ± 11.41	31.61 ± 4.32	24.42 ± 10.55
***Myrtus communis***	Methanol F.	2.02 ± 0.69	38.70 ± 9.90	19.43 ± 0.21
***Melissa officinalis***	Crude E.	33.01 ± 4.32	3.20 ± 1.73	11.32 ± 10.36
***Melissa officinalis***	Chloroform F.	2.89 ± 2.80	84.29 ± 31.20	14.39 ± 3.67
***Melissa officinalis***	Methanol F.	64.57 ± 5.12	27.42 ± 6.20	67.66 ± 8.87
***Hypericum perforatum***	Crude E.	33.78 ± 3.31	17.81 ± 0.79	21.99 ± 4.20
***Hypericum perforatum***	Chloroform F.	45.21 ± 12.37	44.95 ± 11.18	74.80 ± 3.92
***Hypericum perforatum***	Methanol F.	3.04 ± 3.27	48.61 ± 8.44	62.56 ± 6.23
***Tilia platyphyllos***	Crude E.	10.44 ± 3.85	3.89 ± 1.57	48.37 ± 22.95
***Tilia platyphyllos***	Chloroform F.	77.17 ± 5.54	23.35 ± 10.03	71.12 ± 9.35
***Tilia platyphyllos***	Methanol F.	90.78 ± 7.56	45.77 ± 3.50	41.79 ± 17.84
***Salix alba***	Crude E.	88.27 ± 9.67	78.03 ± 4.47	50.48 ± 11.32
***Salix alba***	Chloroform F.	98.71 ± 17.46	64.78 ± 6.56	52.35 ± 14.25
***Salix alba***	Methanol F.	68.60 ± 10.69	38.46 ± 3.80	34.24 ± 9.92
***Camellia sinensis***	Crude E.	16.03 ± 0.86	30.29 ± 13.24	57.43 ± 10.70
***Camellia sinensis***	Chloroform F.	80.67 ± 18.75	95.54 ± 15.29	29.40 ± 10.98
***Camellia sinensis***	Methanol F.	16.24 ± 0.39	11.36 ± 0.85	74.05 ± 15.81
***Camellia sinensis*(fermented)**	Crude E.	99.40 ± 16.05	80.63 ± 19.28	57.27 ± 4.49
***Camellia sinensis*(fermented)**	Chloroform F.	90.46 ± 16.83	57.23 ± 36.94	41.18 ± 11.35
***Camellia sinensis*(fermented)**	Methanol F.	90.58 ± 9.05	58.85 ± 2.59	53.53 ± 5.45
**Amantadine hydrochloride**	**–**	83.54 ± 3.16	42.25 ± 4.76	43.66 ± 5.76
**Oseltamivir carboxylate**	**–**	94.78 ± 0.40	89.63 ± 0.81	87.33 ± 3.33

In co-penetration treatments, *G. glabra* (methanol fraction;0.86 ± 0.29), *M. officinalis* (methanol fraction; 0.81 ± 0.05), *T. platyphyllos* (methanol fraction; 0.81 ± 0.05), *T. platyphyllos* (chloroform fraction; 0.72 ± 0.04), *S. alba* (crude extract; 0.80 ± 0.07), *S. alba* (methanol extract; 0.66 ± 0.07), *S. alba* (chloroform extract; 0.87 ± 0.11), *C. sinensis* (chloroform extract; 0.74 ± 0.13), *C. sinensis* (fermented crude extract; 0.87 ± 0.11), *C. sinensis* (fermented methanol fraction; 0.80 ± 0.04), *C. sinensis* (fermented chloroform fraction; 0.81 ± 0.12); in pre-penetration treatments, *G. glabra* (crude extract; 1.05 ± 0.10), *M. officinalis* (chloroform fraction; 1.01 ± 0.31), *S. alba* (crude extract; 0.73 ± 0.03), *C. sinensis* (chloroform fraction; 0.81 ± 0.16), *C. sinensis* (fermented crude extract; 0.74 ± 0.13), *C. sinensis* (fermented chloroform fraction; 0.79 ± 0.25); and in post-penetration treatments, *M. communis* (crude extract; 0.88 ± 0.05), *M. officinalis* (methanol fraction; 0.84 ± 0.09), *H. perforatum* (methanol fraction; 0.79 ± 0.06), *H. perforatum* (chloroform fraction; 0.915 ± 0.038), *T. platyphyllos* (chloroform fraction; 0.68 ± 0.07), *C. sinensis* (methanol fraction; 0.70 ± 0.11) showed the highest cell viability and protection against virus cytopathic effects.

### Dose-dependent response

The test was repeated twice. Some of the extracts and fractions showed RBC precipitation (HI+) until a certain dilution which showed dose-dependent responses for their HA physical interaction. But some other samples showed RBC precipitation (HI+) in all dilutions which is indicative of HA physical interaction in all dilutions (Table 7).

**Table 7:** Dose-dependent response

Plant	Extracts or Fractions	Dilution to give HI+
***Glycyrrhiza glabra***	Crude E.	4th
***Glycyrrhiza glabra***	Chloroform F.	4th
***Glycyrrhiza glabra***	Methanol F.	4th
***Myrtus communis***	Crude E.	5th
***Myrtus communis***	Chloroform F.	4th
***Myrtus communis***	Methanol F.	4th
***Melissa officinalis***	Crude E.	–
***Melissa officinalis***	Chloroform F.	–
***Melissa officinalis***	Methanol F.	3rd
***Hypericum perforatum***	Crude E.	3rd
***Hypericum perforatum***	Chloroform F.	5th
***Hypericum perforatum***	Methanol F.	3rd
***Tilia platyphyllos***	Crude E.	**–**
***Tilia platyphyllos***	Chloroform F.	**–**
***Tilia platyphyllos***	Methanol F.	**–**
***Salix alba***	Crude E.	**–**
***Salix alba***	Chloroform F.	**–**
***Salix alba***	Methanol F.	**–**
***Camellia sinensis***	Crude E.	**–**
***Camellia sinensis***	Chloroform F.	**–**
***Camellia sinensis***	Methanol F.	**–**
***Camellia sinensis*(fermented)**	Crude E.	**–**
***Camellia sinensis*(fermented)**	Chloroform F.	**–**
***Camellia sinensis*(fermented)**	Methanol F.	**–**
**Amantadine hydrochloride**	**–**	3rd
**Oseltamivir carboxylate**	**–**	3rd

### Preliminary phytochemical analysis results

The existence of secondary metabolites was investigated by different preliminary analyses for the crude extracts, and chloroform and methanol fractions of five potent anti-influenza virus plants including *G. glabra*, *M. communis*, *M. officinalis*, *S. alba* and *C. sinensis* (fermented). Phytochemical analysis data (Table 8) confirmed the presence of alkaloids, cardiac glycosides, tannins, flavonoids, triterpenoids and steroids in the active crude extracts of *G. glabra*, *M. officinalis* and *S. alba*. The effective chloroform fractions of *M. communis* and *C. sinensis* (fermented) were rich in alkaloids, cardiac glycosides, triterpenoids and steroids. Methanol fractions of *M. communis* and *M. officinalis* with potential antiviral activities against influenza virus contained high amounts of flavonoids, tannins, triterpenoids and steroids.

**Table 8:** Preliminary phytochemical analysis of crude extracts and different fractions of five potential plants with anti-influenza A activity

Phytochemical tests		*Glycyrrhiza* *glabra*			*Myrtus commonis*			*Melissa* *officinalis*			*Salix* *alba*			*Camellia sinensis* (fermented)		
		C E	C F	M F	C E	C F	M F	C E	C F	M F	C E	C F	M F	C E	C F	M F
**Alkaloids**	Dragendorff’s Test	_	_	_	+	*+*	_	+	+	_	+	_	+	+	+	_
	Hager’s Test	_	_	_	+	_	+	+	+	_	+	+	_	+	+	_
**Cardiac Glycosides**	Keller-killiani	+	_	+	+	+	_	+	+	_	+	_	+	+	+	_
**Tannins**	Ferric Chloride Test	+	_	+	+	_	+	_	_	_	+	_	+	+	_	+
	Gelatin test	+	+	+	+	_	+	_	_	_	+	_	+	+	_	+
**Flavonoids**	Shinoda Test	+	_	+	+	_	+	+	_	+	+	_	+	+	_	+
**Steroids and Triterpenoids**	Salkowski test	+	+	_	+	+	+	+	_	+	+	+	_	+	+	+
	Libermann-Buchard test	_	_	_	_	+	_	+	+	+	_	+	_	_	+	_
**Saponin**	Foam test	_	_	_	_	_	_	_	_	_	_	_	_	_	_	_

*CE* Crude Extract, *CF* Chloroform Fraction, *MF* Methanol Fraction; +: present, −:absent

## Discussion

Medicinal plants have progressively been noticed as suitable alternatives to the synthetic antiviral agents [22–25]. In the current research, based on the antiviral properties of medicinal plants against IAV and other viruses, and their traditional and folklore usage in Iran, the antiviral efficacy of the crude extracts, and chloroform and methanol fractions of some Iranian native medicinal plants including *G. glabra, M. communis, M. officinalis, H. perforatum, T. platyphyllos, S. alba*, and *C. sinensis* (fermented and non-fermented) were evaluated against IAV with more details.

Previous investigation revealed that glycyrrhizin derived from the rhizomes of *G. glabra* has protective effects against IAV by induction of interferon [26]. In addition, other compounds of licorice showed significant inhibition on influenza A neuraminidase in a computer-based approach [27]. The effects of polyherbal formula containing licorice were confirmed for the prevention and treatment of influenza-like syndrome, clinically [28, 29].

*M. officinalis* essential oil could inhibit avian influenza virus (H9N2) through various replication cycle steps especially direct interaction with the virus particles [30]. Also, its extract demonstrated a significant anti-influenza effect against H1N1 strain of influenza virus [31].

The extract of *H. perforatum* showed anti-IAV effect both in vitro and in vivo. The EC_50_ of the extract was 40 μg/ml against IAV while its CC_50_ in MDCK cell line was 1.5 mg/ml [32]. In an experiment, it was observed that *H. perforatum* extract had significant efficacy for the treatment of mice infected with IAV [32]. In another study an opposite response occurred. The consumption of oral *H. perforatum* extract in the mice infected with influenza A virus, enhanced transcription of the suppressor of cytokine signaling 3 (SOCS3) and led to the impaired immune defense and higher mortality [33].

The anti-influenza activity of green tea (*Camellia sinensis*) against H1N1 virus was equivalent to green tea by-products (EC_50_ equal to 6.72 and 6.36 μg/ml, respectively). Also, hexane-soluble and ethyl acetate-soluble fractions of green tea by-products possessed strong anti-influenza activity in chickens [34]. The other studies demonstrated that dimeric polyphenol molecules in green tea display more potent antiviral effects against both influenza A and B viruses than monomers. In addition, the existance of C-4′ hydroxyl group in the B ring of planar flavonols is necessary for the anti-influenza B virus activity [35, 36]. It was confirmed clinically that formulations containing *C. sinensis* or green tea metabolites including catechines and theanine could prevent influenza infection [37, 38].

According to the results, in terms of the selectivity index (SI), the extracts and fractions of all tested herbs were considered safe for the antiviral treatments except chloroform fraction of *G. glabra*. Its SI value was the same as conventional drugs. In addition, *C. sinensis*, *S. alba*, *H. perforatum* and *M. officinalis* were categorized as the safest plants in cellular studies.

In HI test, the extracts of *T. platyphyllos*, *S. alba* and *C. sinensis* showed RBC precipitation in all tested dilutions which indicates the strong physical interaction of these compounds with the HA surface glycoprotein of the virus. However, amongst the others, methanol fraction of *M. officinalis* showed the weakest interaction (3rd dilution), and on the opposite side *M. communis* and *H. perforatum* showed stronger interaction (5th dilution). The results of this study confirmed dose-dependent response for most of the extracts and fractions. In a previous research, the formation of complexes between tannins and proteins was confirmed [39]. Also it was demonstrated that antiviral inhibitory effects of hydrolyzable tannins were related to the intractions blocking between viral glycoproteins and cell surface glycosaminoglycans (GAGs) [40]. It is interesting that in our experiment, *M. officinalis*, unlike the others, did not contain any tannin which justificated the weakest intraction.

With a thorough scrutiny on the Tables 3, 4, 5 and 6 and comparing their results with the SI values in Table 2 and the fact that SI values higher than 3 are safe compounds, it was concluded that except one almost all tested samples were safe but not effective against IAV titer.

Moreover, General Linear Model (GLM) analysis which estimated marginal means of all the respective values for different exposure ways (combined treatments) confirmed all the outcomes. The data are shown in the Supplementary Figs. 1, 2, 3 and 4. Therefore, most of the above mentioned plant species might be promising alternatives to decrease flu unfavorable effects by affecting the viral and cellular receptors. The data were considerable because the conventional antiviral drugs; amantadine and oseltamivir showed promising effects against virus infection, however, growing drug resistance has caused a significant challenge [41, 42].

The phytochemical analysis of the potent anti-IAV extracts/fractions demonstrated that they were rich in flavonoids, tannins, triterpenoids and steroids. Natural products have different mechanisms against viral infections from interfering with entry, transcription, replication and translation of the virus, nuclear export of the virus, viral assembly, packing and budding to enhance the host responses [43].

There were many reports that showed flavonids act as anti-IAV compounds with various mechanisms [44, 45]. Flavonoids are natural phenolic compounds of plants with potent antioxidant and antiviral properties. They can help viral-infected cells to fix their biochemical imbalance resulting from oxidative stress [46]. Also they have shown potential inhibition on the neuraminidase active site of influenza virus. The potency of NA blocking reduced from aurones to flavon(ol)es, isoflavones, flavanon(ol)es and flavan(ol)es, respectively. The structure activity relationship (SAR) studies of flavonoids against influenza virus demonstrated that the presence of 7-OH, 4′-OH, C_2_ = C_3_ and C_4_ = O functionalities were necessary, but the existance of a sugar group reduced the effects [47, 48].

Triterpenoids and steroids are natural components elucidated from plants and other organisms which have various biological activities including antiviral activities. Mechanistic studies revealed triterpenoids bind tightly to the viral hemagglutinin (HA) and disrupt the attachment of viruses to the cell receptors [49–51].

Influenza virus has two envelope glycoproteins named hemagglutinin (HA) and neuraminidase (NA). The binding of hemagglutinin to sialic acid residues of the host cells is a key step for initiating the IAV infection. The role of NA is facilitating the movement of virus from infection sites to the respiratory tract [52, 53]. The presence of flavonoids, tannins, steroids and triterpenoids together in the potent anti-influenza extracts and fractions of this study, covers the inhibitory effects on both the HA and NA may be a reason for such significant effects. It is good to note that the virus strain used in this study, A/PR/8/34, is not pathogenic to humans and may not be the best model for these types of studies. However, this strain is generally used because it provides acceptable oucomes comparable to the pathogenic strains and is also safe for the users.

## Conclusions

The emergence of new strains of the influenza A virus makes us think about innovative strategies for the development of new drugs with improved antiviral effects, higher safety and better tolerability. Research focusing on traditional herbs as complementary therapies or preventive medicine is becoming more attractive. Medicinal plants have been used for various ailments, particularly infectious diseases. The current study indicated that treatment of IAV with the selected extracts and fractions reduced the hemagglutination activity of the virus, which may result from the physical interaction of the samples with virus hemagglutinin. Based on this scientific confirmation, the selected plants may have the capacity to ease the symptoms and burden of flu. The next focus of this study will be the purification of pure compounds responsible for the bioactivity against IAV infection as well as their mode of action. Recommendations are proposed for strategizing the future role and place of medicinal plants in prevention and treatment of influenza and other infectious diseases in national, regional and international health policies and programs.

## Supplementary Information

**Additional file 1: Supplementary Fig. 1**. Estimated marginal means of Log HA titer. This graph shows the Log HA titer levels analyzed by GLM. *Glycyrrhiza glabra* crude extract (1), *Glycyrrhiza glabra* methanol fraction (2), *Glycyrrhiza glabra* chloroform fraction (3), *Myrtus communis* crude extract (4), *Myrtus communis* methanol fraction (5), *Myrtus communis* chloroform fraction (6), *Melissa officinalis* crude extract (7), *Melissa officinalis* methanol fraction (8), *Melissa officinalis* chloroform fraction (9), *Hypericum perforatum* crude extract (10), *Hypericum perforatum* methanol fraction (11), *Hypericum perforatum* chloroform fraction (12), *Tiliatilia platyphyllos* crude extract (13), *Tilia platyphyllos* methanol fraction (14), *Tilia platyphyllos* chloroform fraction (15), *Salix alba* crude extract (16), *Salix alba* methanol fraction (17), *Salix alba* chloroform fraction (18), *Camellia sinensis* crude extract (19), *Camellia sinensis* methanol fraction (20), *Camellia sinensis* chloroform fraction (21), *Camellia sinensis* fermented crude extract (22), *Camellia sinensis* fermented methanol fraction (23), *Camellia sinensis* fermented chloroform fraction (24), Amantadine hydrochloride (25), Oseltamivir carboxylate (26), IAV (27), 1 (blue): Co-penetration, 2(green): Pre-penetration, 3 (brown): Post-penetration.

**Additional file 2: Supplementary Fig. 2**. Estimated marginal means of Log HA decrement. This graph shows the decrement levels in Log HA titers analyzed by GLM.

**Additional file 3: Supplementary Fig. 3**. Estimated marginal means of cell viability. This graph shows the ODs of the cell viability test analyzed by GLM.

**Additional file 4: Supplementary Fig. 4**. Estimated marginal means of the percentage of protection. This graph shows the protection of the extracts on the cell viability analyzed by GLM.

## Data Availability

All data and materials are contained and descr ibed within the manuscript. The datasets used and/or analyzed during the current study are available from the corresponding author on reasonable request.

## Abbreviations

ANOVA: Analysis of variance
CCID_50_: Cell culture infectious dose 50
CC_50_: 50% cytotoxic concentration
cRBCs: Chicken red blood cells
DMEM: Dulbecco’s Modified Eagle’s Medium
EC_50_: 50% effective concentration
FBS: Fetal Bovine Serum
GLM: General Linear Model
HA: Hemagglutination Assay
HI: Hemagglutination Inhibition Assay
IAV: Influenza A Virus
MDCK: Madin Darby Canine Kidney
MTT: [3-(4,5-dimethyl-2-thiazolyl)-2,5-diphenyl-2H-tetrazolium bromide]
OD: Optical Density
SI: Selectivity Index
Trypsin-TPCK: Tosylamide Phenylethyl Chloromethyl Keton-treated Trypsin
