# Supplementary figures and images for: Potential antiviral effects of some native Iranian medicinal plants extracts and fractions against influenza A virus
Source: BMC Complement Med Ther. 2021 Oct 1;21:246. doi: 10.1186/s12906-021-03423-x (PMC8485427; doi:10.1186/s12906-021-03423-x)

**
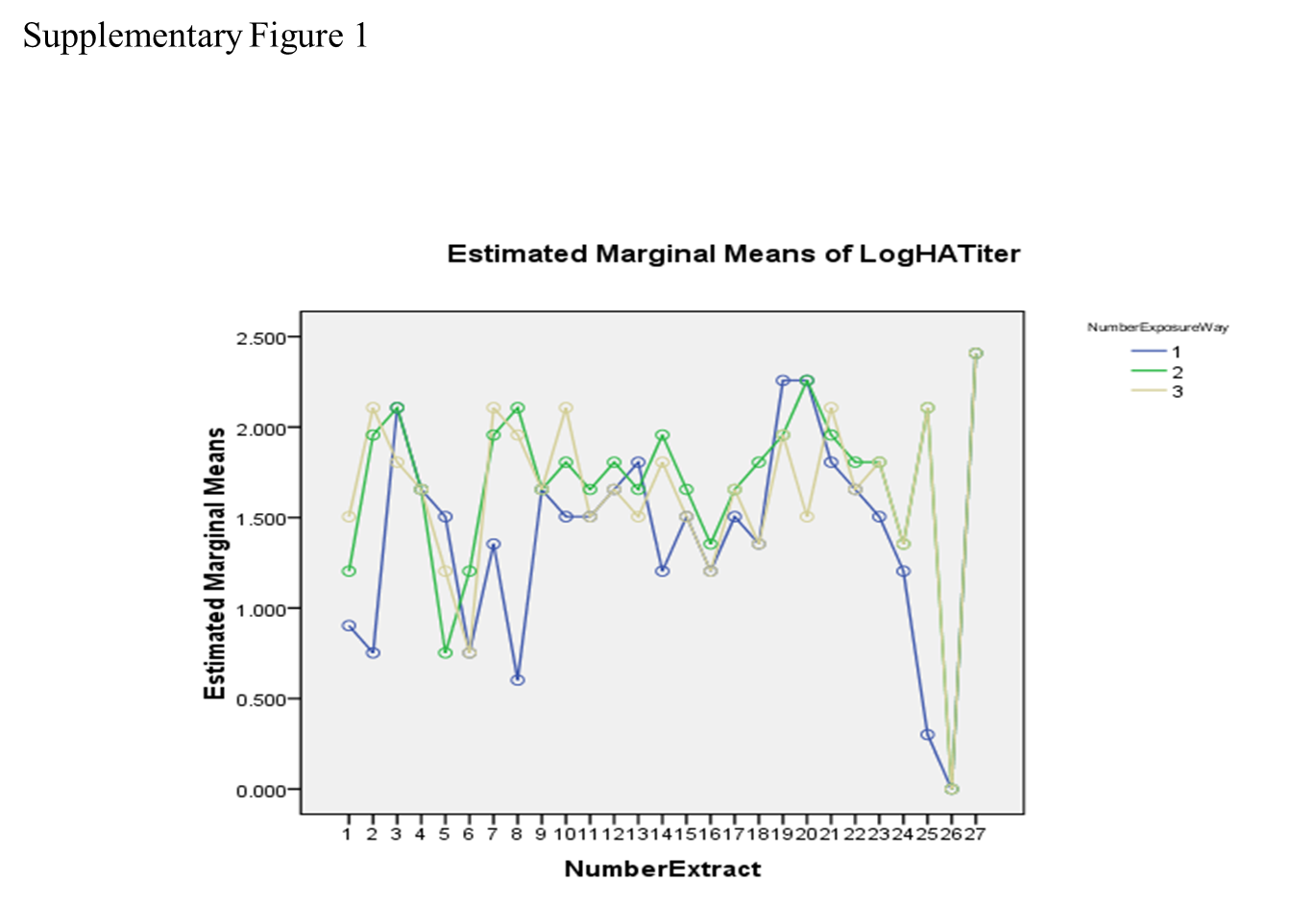
**

Supplement: Supplementary file 1 — Additional file 1: Supplementary Fig. 1. Estimated marginal means of Log HA titer. This graph shows the Log HA titer levels analyzed by GLM. Glycyrrhiza glabra crude extract (1), Glycyrrhiza glabra methanol fraction (2), Glycyrrhiza glabra chloroform fraction (3), Myrtus communis crude extract (4), Myrtus communis methanol fraction (5), Myrtus communis chloroform fraction (6), Melissa officinalis crude extract (7), Melissa officinalis methanol fraction (8), Melissa officinalis chloroform fraction (9), Hypericum perforatum crude extract (10), Hypericum perforatum methanol fraction (11), Hypericum perforatum chloroform fraction (12), Tiliatilia platyphyllos crude extract (13), Tilia platyphyllos methanol fraction (14), Tilia platyphyllos chloroform fraction (15), Salix alba crude extract (16), Salix alba methanol fraction (17), Salix alba chloroform fraction (18), Camellia sinensis crude extract (19), Camellia sinensis methanol fraction (20), Camellia sinensis chloroform fraction (21), Camellia sinensis fermented crude extract (22), Camellia sinensis fermented methanol fraction (23), Camellia sinensis fermented chloroform fraction (24), Amantadine hydrochloride (25), Oseltamivir carboxylate (26), IAV (27), 1 (blue): Co-penetration, 2(green): Pre-penetration, 3 (brown): Post-penetration. [file 12906_2021_3423_MOESM1_ESM.docx]

**
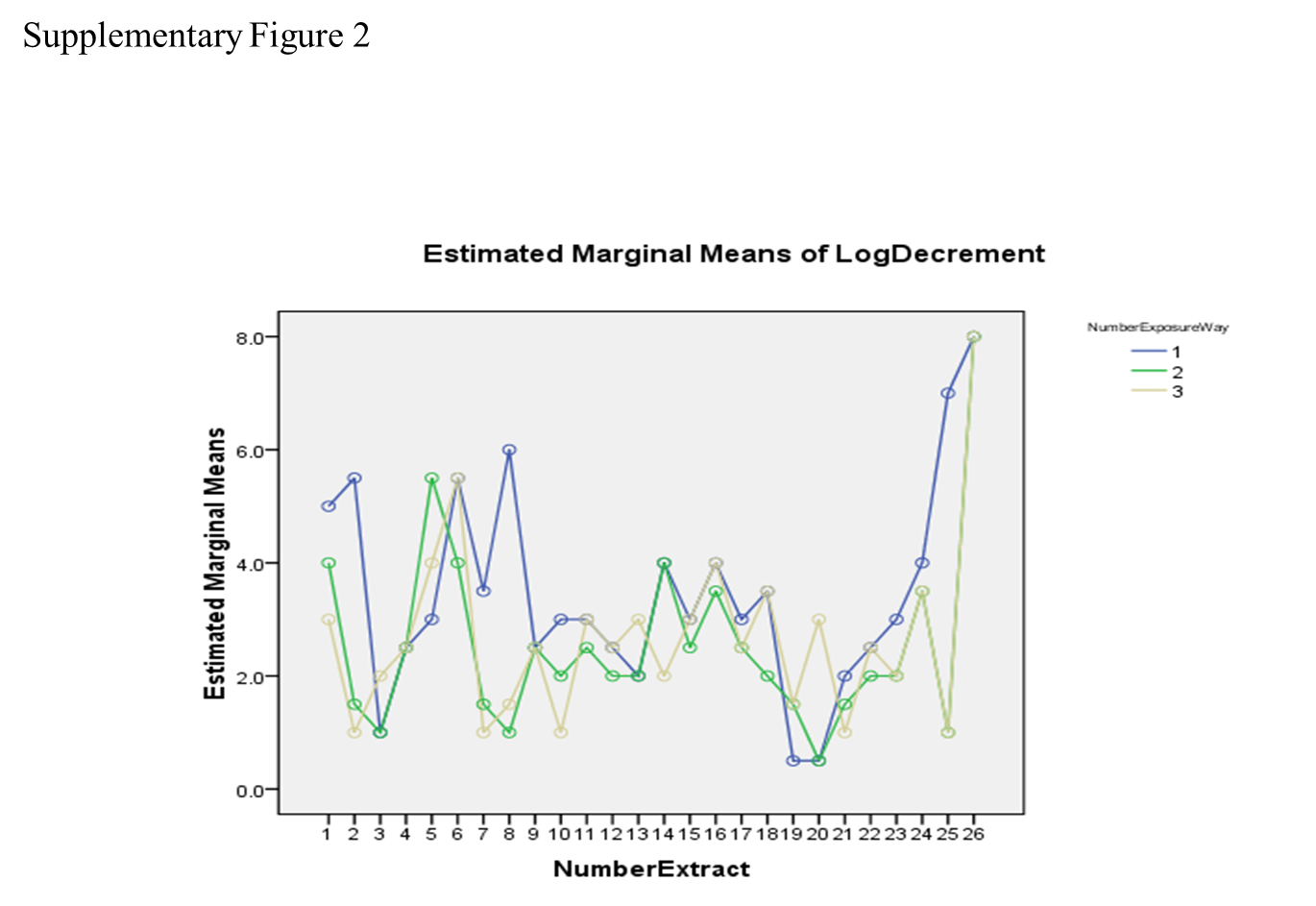
**

Supplement: Supplementary file 2 — Additional file 2: Supplementary Fig. 2. Estimated marginal means of Log HA decrement. This graph shows the decrement levels in Log HA titers analyzed by GLM. [file 12906_2021_3423_MOESM2_ESM.docx]

**
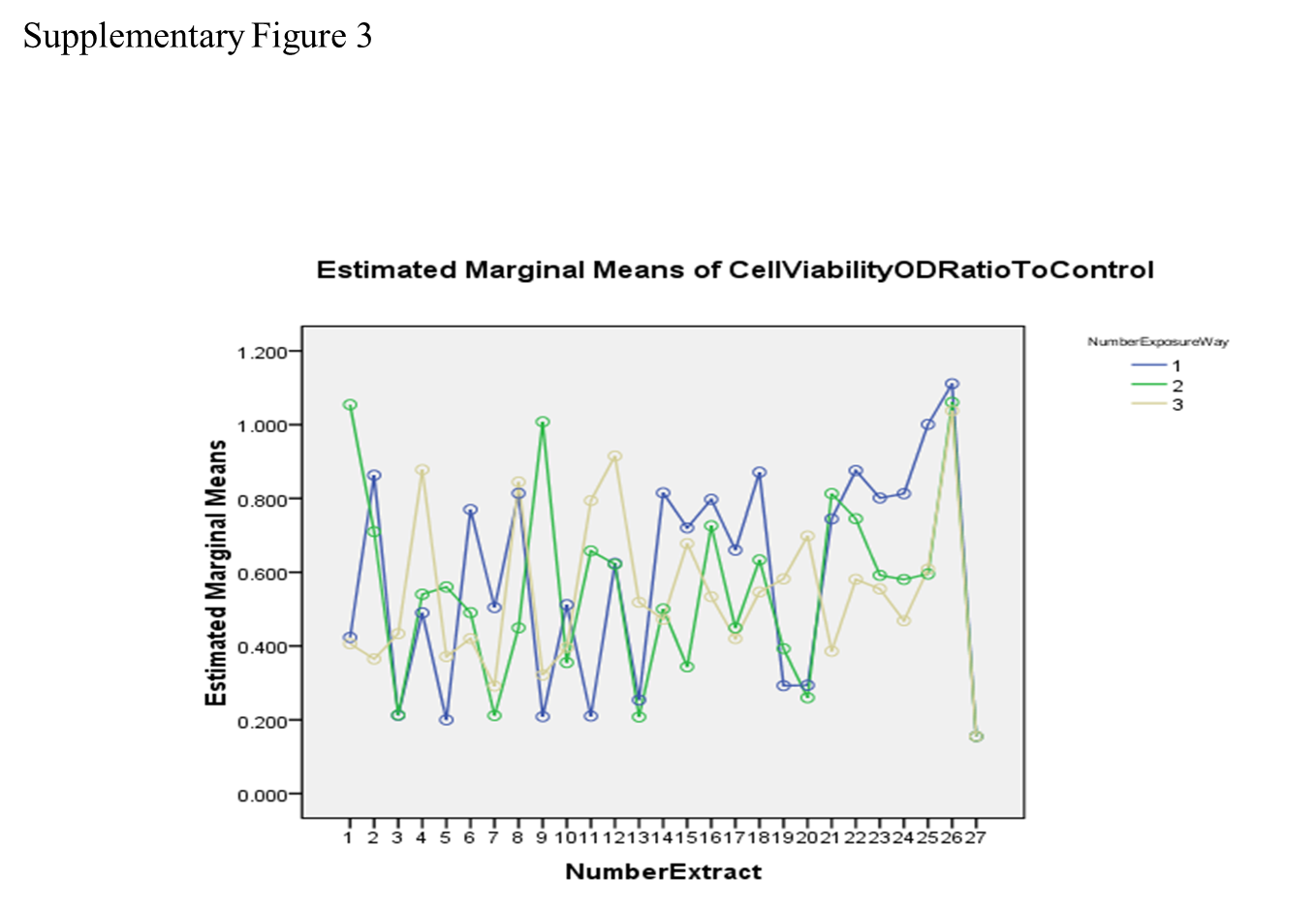
**

Supplement: Supplementary file 3 — Additional file 3: Supplementary Fig. 3. Estimated marginal means of cell viability. This graph shows the ODs of the cell viability test analyzed by GLM. [file 12906_2021_3423_MOESM3_ESM.docx]

**
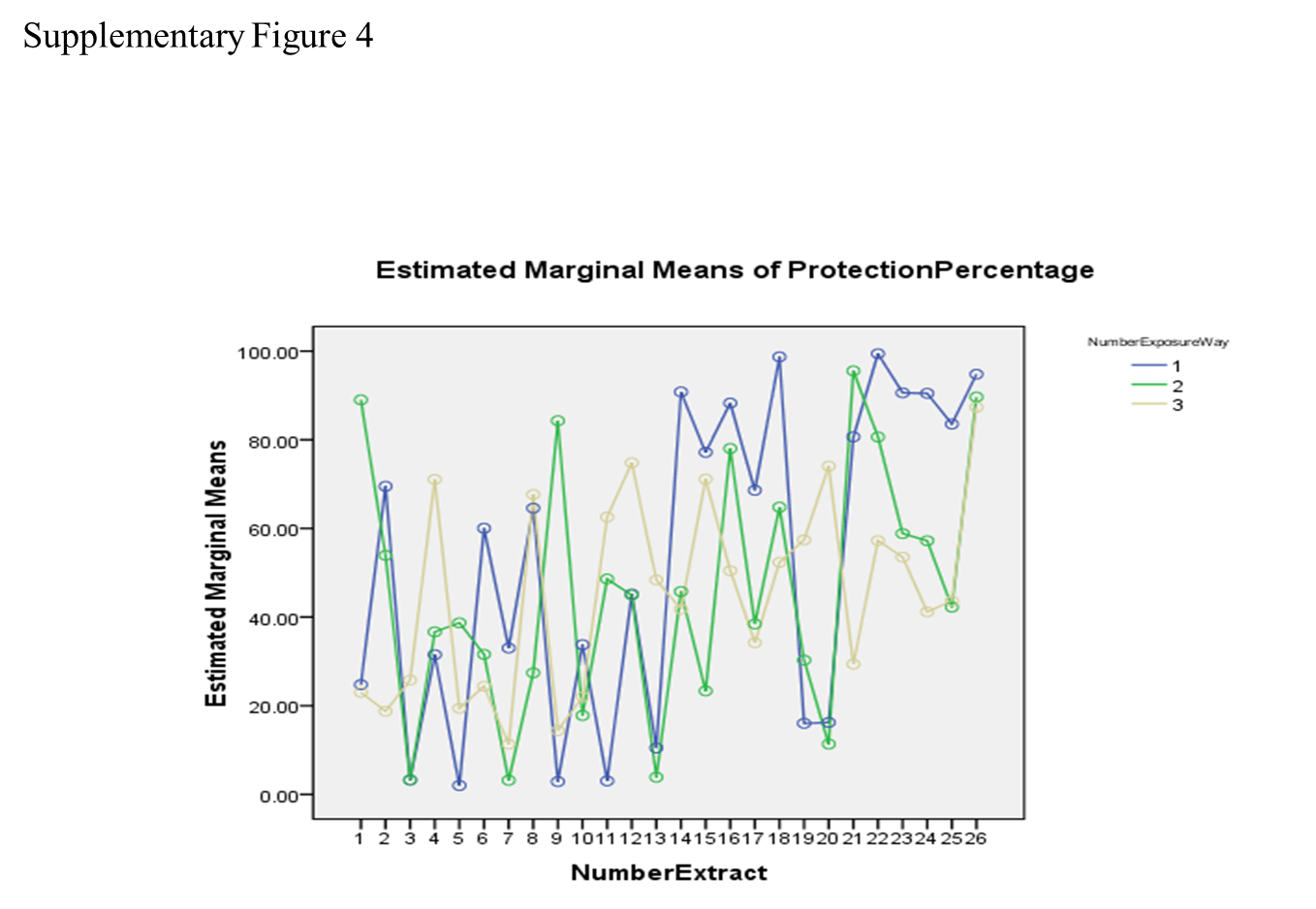
**

Supplement: Supplementary file 4 — Additional file 4: Supplementary Fig. 4. Estimated marginal means of the percentage of protection. This graph shows the protection of the extracts on the cell viability analyzed by GLM. [file 12906_2021_3423_MOESM4_ESM.docx]
